# Coupling between regional aortic pulse wave velocity and luminal diameters in patients with thoracic aortic aneurysm: initial results from the ACTA study

**DOI:** 10.1186/1532-429X-15-S1-P236

**Published:** 2013-01-30

**Authors:** Eleanore Kroner, Jos J Westenberg, Lucia JM Kroft, Niels J Brouwer, Pieter J van den Boogaard, Arthur Scholte

**Affiliations:** 1 Cardiology, LUMC, Leiden, Netherlands; 2 Radiology, LUMC, Leiden, Netherlands

## Background

Thoracic aortic aneurysm (TAA) is life-threatening and requires close follow-up to prevent aortic dissection and/or rupture. Aortic wall stiffness and luminal size are considered to be coupled. Regional aortic wall stiffness in patients with TAA is unknown. Evaluation of regional stiffness may supplement monitoring for prediction of progressive aortic dilatation. Aortic Pulse Wave Velocity (PWV), a marker of vascular stiffness, can be assessed regionally using in-plane two-directional velocity-encoded (VE) MRI. The purpose of this study was to evaluate coupling between regional PWV and aortic luminal diameter in patients with TAA.

## Methods

In 40 TAA patients (mean age 59±13years, 28 male) regional aortic diameters and regional PWV were assessed by 1.5T MRI (Philips). Marfan's disease was excluded. A fast gradient-echo T1-weighted contrast-enhanced MR angiogram of the full aorta was obtained by first pass imaging 25 mL contrast bolus Dotarem (Guerbet) with molarity 0.5 mmol/mL, infused at 2mL/s. Five regional aortic segments were evaluated: ascending aorta (S1), aortic arch (S2), thoracic descending aorta (S3), suprarenal (S4) and infrarenal abdominal aorta (S5). Regional PWV was obtained from multi-slice 2-directional VE MRI, covering the full aorta by 3 double-oblique sagittal slices with in-plane velocity-sensitivity 150cm/s. Regional PWV was determined by wave propagation analysis and calculated as segment length divided by the transit-time of the flow velocity wave propagating through this segment. The incidence of increased diameter (compared to normal values in ref [1]) and increased PWV (compared to age-related normal values in ref [2]) was determined for all aortic segments.

## Results

**Figure 1:**
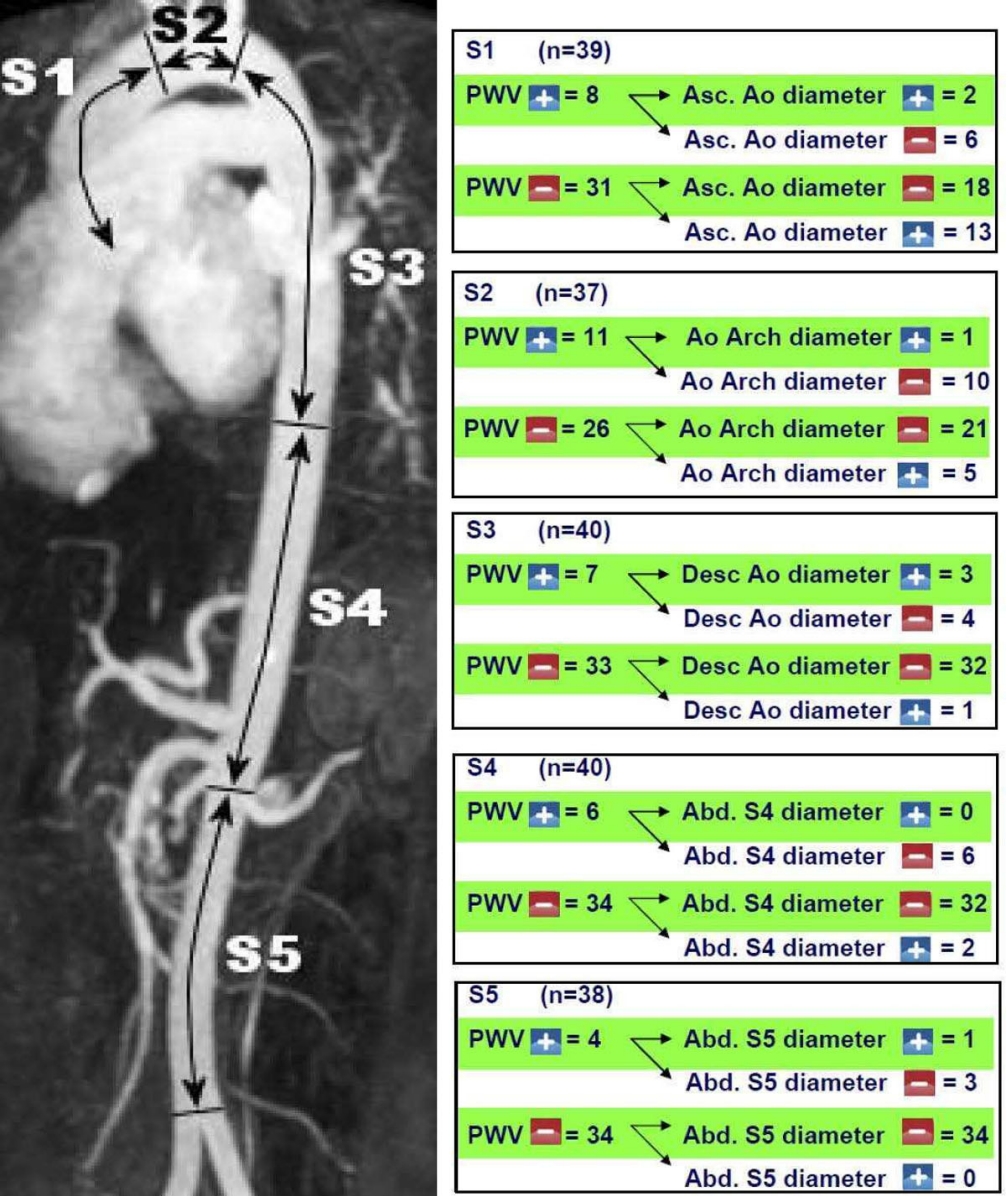
Coupling between regional PWV and regional aortic luminal diameter in five aortic segments.

Mean diameter was 44±5 mm for the aortic root and 39±5 mm for the ascending aorta. Regional PWV was increased compared to age-related normal values in 36 (19%) aortic segments (S1 8 cases, S2 11 cases, S3 7 cases, S4 6 cases, S5 4 cases). Regional aortic diameter was increased in 28 (14%) segments (S1 15 cases, S2 6 cases, S3 4 cases, S4 2 cases, S5 1 cases). In Figure 1, results per segment are given. Table 1 shows sensitivity, specificity and predictive value for regional PWV testing for predicting aortic luminal dilatation TAA patients.

**Table 1:** Sensitivity, specificity, positive and negative predictive value for regional PWV testing in predicting regional aortic dilatation in TAA patients.

Segment	sensitivity	specificity	positive predictive value	negative predictive value
**S1 (n=39)**	2/15	18/24	2/8	18/31
**S2 (n=37)**	1/6	21/32	1/11	21/26
**S3 (n=40)**	3/4	32/36	3/7	32/33
**S4 (n=40)**	0/2	32/38	0/6	32/34
**S5 (n=38)**	1/1	34/37	1/4	34/34

## Conclusions

Normal regional PWV assessed in the full aorta with in-plane VE MRI demonstrates absence of increased luminal diameter, with high specificity and negative predictive value in the descending thoracic to abdominal aorta and moderate results in the ascending aorta and aortic arch. Increased regional PWV may be a precursor to aortic luminal dilatation, but normal regional PWV rules out disease.

## Funding

None
